# The prevalence and antibiotic susceptibility of *Staphylococcus* spp. on ocular surfaces of fighting bulls (*Bos indicus*) in Thailand

**DOI:** 10.14202/vetworld.2022.2922-2928

**Published:** 2022-12-24

**Authors:** Dennapa Saeloh Sotthibandhu, Saowakon Indoung, Husna Niwasawat, Jiradchaya Chaiboon, Nattakan Sungsorn, Nu-issana Longji, Kittipol Polya, Chayanee Noosak, Stefan Schwarz, Tanawan Soimala

**Affiliations:** 1 Faculty of Medical Technology, Prince of Songkla University, Songkhla 90110, Thailand; 2 Faculty of Veterinary Science, Prince of Songkla University, Songkhla 90110, Thailand; 3 Thunderbolt fighting Bull Clinic, Songkhla 90110, Thailand; 4 Department of Veterinary Medicine, Institute of Microbiology and Epizootics, Centre for Infection Medicine, Freie Universität Berlin, 14163 Berlin, Germany; 5 Veterinary Centre for Resistance Research (TZR), Department of Veterinary Medicine, Freie Universität Berlin, 14163 Berlin, Germany

**Keywords:** antimicrobial susceptibility, conjunctival flora, fighting bulls, *Staphylococcus* spp

## Abstract

**Background and Aim:**

fighting bulls have a high risk of eye injuries, and opportunistic conjunctival bacterial flora may cause subsequent eye diseases. There is little information about the ocular health care of fighting bulls in Thailand. Thus, this study aimed to estimate the prevalence of *Staphylococcus* spp. from the eyes of fighting bulls and investigate their antimicrobial susceptibility.

**Materials and Methods:**

The samples were collected from the right conjunctival sacs of 105 fighting bulls. Biochemical tests and matrix-assisted laser desorption/ionization time-of-flight mass spectrometry were used to identify bacteria to genus and species levels. Antimicrobial susceptibility was tested by agar disk diffusion.

**Results:**

*Staphylococcus* spp. (36.84%, 56/152) were the most detected bacteria. The most prevalent *Staphylococcus* spp. was *Staphylococcus chromogenes* (37.50%, 21/56). The susceptibility test revealed that all isolates were susceptible to sulfamethoxazole/trimethoprim (56/56, 100%) and most were susceptible to chloramphenicol and gentamicin (54/56, 96.43%). The highest resistance rates were seen for tetracycline and doxycycline (23.21%, 13/56) followed by erythromycin (19.64%, 11/56). In addition, *S. chromogenes* isolates were evaluated for their ability to produce biofilms by a quantitative biofilm production assay. A total of 21 isolates exhibited biofilm production, independent of their antimicrobial susceptibility. Three multidrug-resistant isolates were found, including two *Staphylococcus epidermidis* isolates and a single *S. chromogenes* isolate.

**Conclusion:**

As antimicrobial resistant bacteria were detected on the eye surface, veterinarians should always conduct antimicrobial susceptibility testing before using antimicrobial agents. The results from this study will help to improve the standard of eye treatment for fighting bulls in Thailand.

## Introduction

Southern fighting bulls are raised and bred for competition, which is a traditional sport in the south of Thailand [1]. Nowadays, bullfighting is booming, attracting tourists, and stimulating local economies [1]. As the daily routine of the fighting bull is to fight constantly, the health care of these animals is important. This is particularly the case for ocular health because bullfighting is associated with a high risk of causing eye injuries, which may lead to infections [2]. When the eyes get injured, the conjunctival bacterial flora may take the opportunity to induce eye diseases, especially infectious bovine keratoconjunctivitis (IBK) [2–5]. This is the most common ocular disorder in bulls and may be caused by *Moraxella* spp. and *Staphylococcus* spp.; it can be accompanied by severe keratitis [3, 4]. If the treatment is not timely, the patient may suffer from ocular infections, which may increase the severity of the disease and can quickly result in severe and uncontrolled inflammation, leading to the final stage of the disease at which the animal may lose its eyesight [2–5].

Although the bacteria on the ocular surfaces mostly belong to the physiological microbiota, some of them are facultatively pathogenic bacteria that can express antimicrobial resistance and virulence genes [6, 7]. For instance, *Staphylococcus* strains may carry and express the genes called *mecA*, *mecB*, and *mecC*, which code for alternative penicillin-binding proteins that render the use of antimicrobial therapy with b-lactam antibiotics ineffective [6, 7].

Multidrug-resistant (MDR) bacteria in animals, including methicillin-resistant *Staphylococcus pseudintermedius* and *Staphylococcus aureus*, extended-spectrum beta-lactamase-producing *Escherichia coli*, and MDR *Salmonella enterica* subsp. *enterica* serovars, require actions to protect immunocompromised animals and employees of healthcare places [8–10]. *Staphylococcus* spp. frequently produce some of the important virulence traits, such as biofilms and slime layers, which can cause drug resistance [11, 12]. Biofilms increase antimicrobial resistance by the limited diffusion of antimicrobial drugs to bacteria [13]. Slime layers allow bacteria to adhere to protein-coated foreign bodies and basement membranes, initiating infection and growing as biofilms [14].

There is little information in the published literature about the ocular health care of fighting bulls in Thailand. Therefore, this study aimed to estimate the prevalence of the bacterial microbiota in the conjunctival sac of fighting bulls. Once the dominant bacteria were identified, their antimicrobial susceptibility patterns were subsequently investigated.

## Materials and Methods

### Ethical approval

All animal protocols were approved by the Institutional Animal Care and Use Committee, Prince of Songkla University (EC 2564-05-023, June 16, 2021).

### Study period and location

The samples were collected from July 2021 to March 2022 from 105 bulls during routine ocular examinations for both eyes in Songkhla, Thailand.

### Questionnaire and interviews

Data on the description of each animal and the application of antimicrobial agents (systemic and topical) were collected before sampling. Owner interviews were conducted to gather information concerning housing, maintenance, the presence and contact with other animals, the occupation of the owner, and the relationship of the owner with healthcare personnel. The bull owners were also asked to respond to a questionnaire regarding the health status of the bulls. Bulls with healthy condition were subjected to sample collection.

### Sample collection

The samples were collected from 105 bulls during routine ocular examinations of both eyes. The bulls that presented with normal eyes were included in this study. A sterile swab (Yangzhou Chuangxin Medical Device Factory, Jiangsu, China) was moistened with sterile water (Amanta Healthcare Ltd., Gujarat, India). After that, only one swab sample from the right eye was obtained from each bull by placing the sterile moistened swab in the conjunctival sac and rolling it three times. This procedure was performed by the same person (Tanawan Soimala). After sample collection, the specimens were immediately submitted to the Faculty of Veterinary Science, Prince of Songkla University, Songkhla, Thailand, for the primary screening of the bacterial microbiota.

### Ocular and physical examination

To avoid contamination of the sample, the ocular examinations of both eyes were performed after sample collection by an experienced veterinary ophthalmologist. To obtain information to confirm the normality of the eyes, several tests, including external observation, vision evaluation (menace response and obstacle course), reflex tests (dazzle, pupillary light, and palpebral reflexes), and the fluorescent eye stain test, were carried out. Measurement of the intraocular pressure and the Schirmer tear test were performed; the normal ranges are between 15–25 mm/Hg and 15–20 mm/min, respectively [15, 16]. Finally, slit-lamp biomicroscopy was performed to inspect the ocular abnormality of the ocular surfaces. Physical examination, including heart sound, heart rate, lung sound, respiratory rate, capillary refilling time, and temperature measurements, was also performed. Bulls that presented with ocular infections/abnormalities or clinically abnormal health status were excluded from the study.

### Bacterial culture and identification

To investigate the bacterial microbiota in the conjunctival sac, all swabs were streaked onto sheep blood agar (SBA; Clinical Diagnostics Ltd., Bangkok, Thailand) and MacConkey agar plates (Clinical Diagnostics Ltd.) and incubated under aerobic conditions for 24–48 h at 37°C. Isolates with different colony morphologies that had grown on these plates were restreaked onto SBA for further purification. Colonies (≥1 mm) were restreaked onto Tryptic Soy Agar (Clinical diagnostics Ltd.) and incubated under aerobic conditions for 24–48 h at 37°C. Bacterial identification was performed by colony morphology evaluation, hemolysis pattern analysis, Gram staining, catalase testing, and oxidase testing according to standard protocols. All bacteria were transferred to 20% glycerol and frozen at −80°C until use.

Identification to genus and species levels was conducted for all bacteria using a matrix-assisted laser desorption/ionization time-of-flight mass spectrometer (MALDI-TOF MS) (Bruker Daltonik GmbH, Bremen, Germany). A colony of the bacteria was directly smeared on an MTP BigAnchorChip 384 TF target plate (Bruker Daltonik GmbH, Bremen, Germany). One microliter of 70% formic acid was spotted over the bacteria and dried at room temperature(25°C). Thereafter, α-cyano-4-hydroxycinnamic acid, matrix solution, was immediately added to overlay the smear. After air drying at 25°C, MALDI-TOF MS measurement was performed and spectra were generated using a Bruker microflex LT instrument. The MALDI Biotyper 1.1 software (Bruker Daltonik GmbH, Bremen, Germany) was used to find the peak matches against the reference database 1.1.

### Antimicrobial susceptibility testing

The most prevalent bacteria, *Staphylococcus* spp. isolates, were subsequently subjected to conduct antimicrobial susceptibility testing. For this, a single colony was selected, transferred into 0.85% sterile saline, and vortexed. Subsequently, the bacterial suspensions were adjusted to McFarland standard 0.5 (number of bacteria about 1.5 × 10^8^ colony-forming units/mL) according to the recommendation of the Clinical and Laboratory Standards Institute (CLSI) [17]. A sterile cotton swab was placed into the solution and then streaked onto Mueller-Hinton agar (MHA). After 3–5 min, the antimicrobial disks were placed onto the surface of the inoculated MHA plate and incubated at 35°C ± 2°C for 18–24 h in ambient air. After that, the zones of growth inhibition around each disk were measured and compared with the clinical breakpoints mentioned in the CLSI document VET01S [18].

The choice of the antimicrobial agents to be tested was based on (i) whether they exhibited a broad spectrum of activity against both Gram-positive and Gram-negative bacteria and (ii) which antimicrobial agents were frequently used in the ophthalmic routine of cattle in the Veterinary Teaching Hospital, Prince of Songkla University. The antimicrobial disks (all purchased from Clinical Diagnostics Ltd.) included gentamicin (10 μg), enrofloxacin (5 μg), clindamycin (2 μg), erythromycin (15 μg), chloramphenicol (30 μg), oxacillin (1 μg), cefoxitin (30 μg), doxycycline (30 μg), sulfamethoxazole/trimethoprim (23.75/1.25 μg), and tetracycline (30 μg). *Staphylococcus aureus* ATCC^®^ 25923 served as a quality control strain. An isolate was classified as MDR when it exhibited resistance to antimicrobial agents of three or more classes [19, 20].

### Evaluation of biofilm production in clinical isolates of *Staphylococcus chromogenes*

Twenty-one isolates of *S. chromogenes* were quantitatively evaluated for biofilm production using the microtiter plate method described previously [12]. Freshly subcultured isolates of *S. chromogenes* with 0.5 McFarland density were added to a 96-well microtiter plate with Tryptic Soy Broth containing 1% glucose. After 24 h incubation at 37°C, planktonic bacterial cells were removed by washing with sterile phosphate buffer solution (pH 7.2). The biofilm was fixed by adding absolute methanol for 20 min and dried overnight. Adherent cells in each well were stained with 2% Hucker’s crystal violet for 15 min. The excess stain was removed, and the biofilm was washed with distilled water. After drying, 33% acetic acid was added to each well, and the optical density (OD) was measured at 570 nm. Based on the OD values, the isolates were categorized as non-biofilm producers, weak, moderate, or strong biofilm producers, as described previously [12]. Experiments were performed in triplicate and repeated three times.

## Results

**Figure-1:**
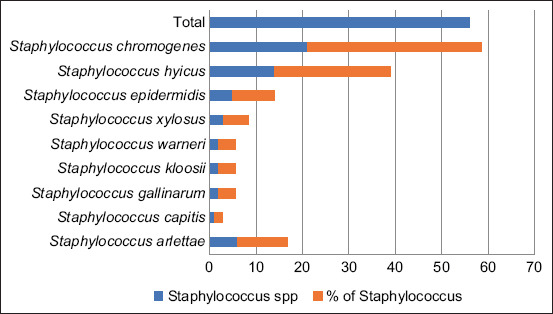
Prevalence of *Staphylococcus spp*. on the conjunctival sac of fighting bulls.

A total of 152 bacterial isolates was obtained from 105 conjunctival samples of fighting bulls (Table-1). *Staphylococcus* spp. (36.84%, 56/152) were the most common bacteria in the conjunctival microbiota in this study. The most prevalent *Staphylococcus* spp. was *S*. *chromogenes* (37.50%, 21/56) followed by *Staphylococcus hyicus* (25.00%, 14/56), *Staphylococcus arlettae* (10.71%, 6/56), *Staphylococcus epidermidis* (8.93%, 5/56), and *Staphylococcus xylosus* (5.36%, 3/56) (Figure-1). *Moraxella* spp. were detected with a prevalence of 1.97% (3/152).

**Table-1:** Prevalence of conjunctival microbiota in the conjunctival sac of fighting bulls.

Bacteria	Prevalence	Percentage
*Staphylococcus* spp.	56	36.84
*Bacillus* spp.	21	13.82
*Rothia* spp.	10	6.58
*Mammaliicoccus sciuri*	10	6.58
*Pseudomonas* spp.	6	3.95
*Acinetobacter* spp.	6	3.95
*Kocuria* spp.	6	3.95
*Brevibacterium* spp.	5	3.29
*Klebsiella pneumoniae*	4	2.63
*Curtobacterium albidum*	4	2.63
*Brachybacterium conglomeratum*	3	1.97
*Moraxella* spp.	3	1.97
*Brevundimonas diminuta*	2	1.32
*Cellulosimicrobium cellulans*	2	1.32
*Corynebacterium casei*	2	1.32
*Trichosporon asahii*	2	1.32
*Micrococcus luteus*	1	0.66
*Aerococcus viridans*	1	0.66
*Chryseobacterium montanum*	1	0.66
*Dermacoccus nishinomiyaensis*	1	0.66
*Enterobacter xiangfangensis*	1	0.66
*Enterococcus casseliflavus*	1	0.66
*Glutamicibacter mysorens*	1	0.66
*Pantoea agglomerans*	1	0.66
*Paracoccus* spp.	1	0.66
*Streptococcus hyovaginalis*	1	0.66
Total	152	100.00

Antimicrobial susceptibility testing of *Staphylococcus* spp. revealed that all isolates were susceptible to sulfamethoxazole/trimethoprim (100%, 56/56). Slightly lower susceptibility rates were found for chloramphenicol and gentamicin (96.43%, 54/56) as well as enrofloxacin (94.64%, 53/56). The highest resistance rate was seen for tetracycline and doxycycline (23.21%, 13/56 each) followed by erythromycin (19.64%, 11/56) (Table-2) [18].

**Table-2:** Antimicrobial susceptibility of *Staphylococcus* spp.

Antimicrobial agents	Antimicrobial susceptibility (number of isolates)					

	Susceptible (%)	Reference range*	Intermediate (%)	Reference range*	Resistant (%)	Reference range*
Cefoxitin*	94.12 (48)	≥25 mm	0 (0)	-	5.88 (3)	≤24 mm
Chloramphenicol	96.43 (54)	≥18 mm	1.79 (1)	13–17 mm	1.79 (1)	≤12 mm
Clindamycin	78.57 (44)	≥21 mm	8.93 (5)	15–20 mm	12.50 (7)	≤14 mm
Doxycycline	75.00 (42)	≥25 mm	1.79 (1)	21–24 mm	23.21 (13)	≤20 mm
Enrofloxacin	94.64 (53)	≥23 mm	3.57 (2)	17–22 mm	1.79 (1)	≤16 mm
Erythromycin	78.57 (44)	≥23 mm	1.79 (1)	14–22mm	19.64 (11)	≤13 mm
Gentamicin	96.43 (54)	≥15 mm	1.79 (1)	13–14 mm	1.79 (1)	≤12 mm
Oxacillin**	80 (4)	≥18 mm	0 (0)	-	20 (1)	≤17 mm
Sulfamethoxazole/ trimethoprim	100 (56)	≥16 mm	0 (0)	11–15 mm	0 (0)	≤10 mm
Tetracycline	75.00 (42)	≥23 mm	1.79 (1)	18–22 mm	23.21 (13)	≤17 mm

* Zone diameter breakpoints according to the Clinical and Laboratory Standards Institute [18].

** According to CLSI, the oxacillin disk test is only applicable to *Staphylococcus epidermidis* isolates. For all other staphylococcal isolates investigated in this study, the cefoxitin disk test is the recommended surrogate test for methicillin resistance

Regarding the most frequently isolated bacteria, *S. chromogenes* isolates were evaluated for their biofilm-producing ability by a quantitative biofilm production assay. A total of 21 isolates exhibited biofilm production. Among them, 16 (76.19%) were classified as weak biofilm producers. Four (19.04%) were classified as moderate biofilm producers and a single isolate (4.76%) was classified as a strong biofilm producer. In this study, the biofilm-producing ability of *S. chromogenes* was not related to any antimicrobial resistance pattern (Table-3).

**Table-3:** Antimicrobial susceptibility of *Staphylococcus chromogenes* and classification of biofilm production.

Isolates	Antimicrobial susceptibility*										Biofilm producers

	GEN	ENR	CLI	ERY	CHL	TET	FOX	DOX	SXT	MDR	
S01	S	S	S	S	S	S	S	S	S	No	Weak
S38	S	S	S	S	S	I	S	R	S	No	Weak
S55	S	S	S	S	S	S	S	S	S	No	Weak
S57	S	S	S	S	S	S	S	S	S	No	Weak
S61	S	S	S	S	S	S	S	S	S	No	Strong
S67	S	S	S	S	S	S	S	S	S	No	Weak
S69	S	S	S	S	S	S	S	S	S	No	Weak
S74	I	S	S	S	S	R	S	R	S	No	Weak
S82	S	S	S	S	S	S	S	S	S	No	Weak
S84	S	S	S	S	S	S	S	S	S	No	Weak
S105	S	S	S	S	S	S	R	S	S	No	Weak
S109	S	I	S	R	S	S	S	S	S	No	Weak
S123	S	S	S	S	S	R	S	R	S	No	Moderate
S127	S	S	I	R	S	R	S	R	S	Yes	Weak
S139	S	S	S	S	S	S	S	S	S	No	Moderate
S150	S	S	S	S	S	S	S	S	S	No	Weak
S160	S	S	S	S	S	R	S	R	S	No	Moderate
S158	S	S	S	S	S	S	S	S	S	No	Moderate
S165	S	S	S	S	S	S	S	S	S	No	Weak
S186	S	S	S	S	S	R	S	R	S	No	Weak
S202	S	S	S	S	S	S	S	S	S	No	Weak

* Antimicrobial agents are abbreviated as follows: GEN=Gentamicin, ENR=Enrofloxacin, CLI=Clindamycin, ERY=Erythromycin, CHL=Chloramphenicol, TET=Tetracycline, FOX=Cefoxitin, DOX=Doxycycline, SXT=Sulfamethoxazole/ trimethoprim, MDR=Multidrug resistant

Three MDR isolates were found, including two *S. epidermidis* isolates and one *S. chromogenes* isolate. Table-4 provides information and the history of the MDR isolates and their antimicrobial resistance profiles.

**Table-4:** Characteristics of MDR bacteria.

Isolates	Bacteria	Medical personnel related	Previous diseases	Previous antibiotics used	Fighting history	Vector prevention	Antibiotic resistance
S80	*Staphylococcus epidermidis*	Yes	Blood parasite infection, FMD	Oxytetracycline (long acting), IM	No	Yes	GEN, CLI, ERY, FOX
S127	*Staphylococcus* *chromogenes*	Yes	Wound infection	Penicillin and streptomycin, IM	Yes	Yes	CLI, ERY, TET/DOX
S193	*Staphylococcus epidermidis*	Yes	Wound infection	Penicillin and streptomycin, IM	No	No	CLI, ERY, TET/DOX, FOX

*Abbreviations of the antimicrobial agents are the same as shown in Table 3; FMD=Foot and mouth disease, IM=Intramuscular injection, MDR=Multidrug resistant, GEN=Gentamicin, CLI=Clindamycin, ERY=Erythromycin, TET=Tetracycline, FOX=Cefoxitin, DOX=Doxycycline

## Discussion

The most frequently detected conjunctival microbiota consisted of Gram-positive bacteria, especially *Staphylococcus* spp. The most prevalent species was *S. chromogenes*, followed by *S. hyicus*. In comparison to the previous studies that investigated conjunctival microbiota from healthy bulls in Mosul and Kufa, Iraq, the most frequently detected isolates were also Gram-positive bacteria, such as *Staphylococcus* spp., *Bacillus* spp., and *Corynebacterium* spp. However, Gram-negative bacteria, for example, *Moraxella bovis* and *E. coli*, were less frequently found [21, 22]. In addition, commensal bacteria from the conjunctival sac of bulls in Urmia, Iran, showed that the most frequently occurring Gram-positive bacteria were *Lactobacillus plantarum*, *S. epidermidis*, *S. aureus*, and *Bacillus cereus* [23]. In another study, *E. coli*, *Proteus mirabilis*, and *Enterobacter aerogenes* (meanwhile reclassified as *Klebsiella aerogenes*) isolates were less frequently detected Gram-negative bacteria [23]. A study about normal microbiota on the ocular surfaces of bulls in Erzurum/Turkey reported that Gram-positive bacteria, such as *Streptococcus* spp., *Staphylococcus* spp., *Bacillus* spp., *Corynebacterium* spp., and *Micrococcus* spp., and Gram-negative bacteria, such as *M. bovis*, *E. coli*, *Neisseria* spp., *Pseudomonas* spp., *Klebsiella pneumoniae*, *Haemophilus* spp., *Acinetobacter* spp., and *Proteus vulgaris*, were frequently isolated [24]. Many articles indicated that the differences in the frequency and the type of the bacteria reflect the variation of the physiological microbiota, which may be impacted by geographic location, weather, season, type of animals, nutrition, anatomic region, and differences in the laboratory techniques applied [25, 26].

Several studies have reported that *M. bovis*, *Moraxella ovis*, *Moraxella bovoculi*, *Mycoplasma bovoculi*, *Chlamydophila* spp., *Staphylococcus* spp., and infectious bovine rhinotracheitis virus are involved in IBK [4, 27, 28]. In this study, *Staphylococcus* spp. were most frequently isolated.

In a case report, *Staphylococcus* spp. was the causing agent of IBK [4]. In the present study, isolates of several potentially pathogenic *Staphylococcus* spp. were identified. Therefore, the ocular surfaces should be constantly observed and monitored for the presence of bacterial infections by animal care personnel. If IBK or other bacterial eye diseases are diagnosed at an early stage, it is likely that the treatment (including suitable antimicrobial agents) will be successful.

Based on our results, sulfamethoxazole/trimethoprim was the antimicrobial agent to which all isolates were susceptible, followed by enrofloxacin and chloramphenicol. The highest resistance rates were seen for tetracycline and doxycycline. Here, all tetracycline-resistant isolates were also resistant to doxycycline. Resistance to the macrolide erythromycin was often detected among *S. arlettae* isolates.

Biofilm production is an important virulence factor for bacteria as it promotes their persistence in the environment. As biofilm-embedded bacteria could be up to 1000 times more tolerant to antimicrobial agents [29], several studies have focused on ways of combatting bacterial biofilms [30–32]. In this study, *S. chromogenes*, the most frequently isolated species, was investigated for its ability to form biofilms and for the relationships between biofilm formation and antimicrobial resistance. All tested isolates of *S. chromogenes* were defined as biofilm producers. Weak biofilm producers were found most frequently, followed by moderate biofilm producers, whereas only a single isolate was a strong biofilm producer. The biofilm production levels were independent of the antimicrobial susceptibility of the bacteria. For example, the strong biofilm producer was susceptible to all antimicrobial agents tested.

Besides, three isolates were defined as MDR bacteria, including two isolates of *S. epidermidis* and one isolate of *S. chromogenes*. At present, there is, to the best of our knowledge, no report that describes the therapeutic challenge of MDR among bacteria from the ocular surfaces of fighting bulls in Thailand. Moreover, there is no established protocol to treat such bacteria in Thailand. Fortunately, there was nothing noted in the initial ophthalmic examination records of the bulls in these three cases regarding an infection by MDR bacteria and a mucopurulent ocular discharge. The criteria used for making the diagnosis of ocular tissue infections included the appearance of mucopurulent ocular discharge accompanied by tissue redness, swelling, and changes in the morphology of the infected tissue as visualized by slit-lamp examination. The three bulls from which the MDR bacteria originated had never visited an animal hospital. However, they suffered from chronic disease and had been treated with antimicrobial agents in a mobile clinic before the sample collection. One study indicated that the use of antimicrobial agents is a risk factor of acquiring infections caused by MDR bacteria [33]. Closely related MDR isolates from colonized animals, environmental sites, and staff members have been reported [34]. In this study, the swabs were taken only from the lower conjunctival sac by the same ophthalmologist and not by the veterinarian and the owner who were in contact with the bulls. It may be suspected that veterinary personnel can also be associated with the dissemination of MDR bacteria. Pulsed-field gel electrophoresis (PFGE) and multilocus sequence typing (MLST) are the methods to (1) determine the genomic relationships of methicillin-resistant staphylococci, (2) investigate whether they are of the same, similar, or unrelated types, (3) examine whether they are likely to have originated from the same source, and (4) determine the possible routes of transmission in the animal population [35, 36]. Zubeir *et al*. [37] reported the same PFGE pattern among 10 methicillin-resistant *S. pseudintermedius* isolated from dogs and cats at one veterinary clinic, whereas the diversity of *Pasteurella multocida* isolates circulating in cattle, sheep, goats, poultry, and pigs has been investigated using MLST [38]. However, since PFGE is often difficult to standardize, it is not the best method for epidemiological investigations [36]. For long-term epidemiological investigations, MLST should be then applied because of its uncomplicated protocol and the possibility of using it with the program developed by Bath University, United Kingdom [39]. The characteristics of this program allow the researchers to analyze evolutionary events and compare them with those found by other laboratories through the belonging sequence task in www.mlst.net. [36]. Unfortunately, PFGE and MLST were not applied to the MDR isolates of these three bulls because of time and financial considerations [36]. Information regarding the use of antimicrobial agents to treat bacterial infections in the conjunctival sac of fighting bulls is scarce, and no single treatment protocol is suitable for ocular infections. Therefore, the treatment protocol should be adapted to the individual patient. In addition, many aspects should be considered, for instance, the antimicrobial susceptibility profile of the bacterial isolates, the severity and location of the infection, and the presence of an underlying disease. Moreover, the risk of acquiring further resistant bacteria needs to be considered not only as a health issue for the affected animal but also with regard to the spread of resistant bacteria to other animals or humans.

## Conclusion

As antimicrobial-resistant bacteria have been found on the ocular surface, the veterinarians should always conduct antimicrobial susceptibility testing. In this study, three isolates of staphylococcal bacteria were confirmed as MDR. Therefore, further studies should investigate the source of MDR using molecular microbiology methods. The results from this study may be of major relevance to veterinary practitioners and veterinary ophthalmologists and will improve the standard of treatment for animal eye diseases in Thailand.

## Authors’ Contributions

DSS and TS: Conceptualization, DSS, SI, HN, JC, NS, NL, and TS: Methodology, HN, JC, NS, NL, KP, and TS: Data collection, DSS, SI, SS, and TS: Validation, DSS, HN, JC, NS, NL, SS, and TS: Formal analysis, DSS, SI, CN, and TS: Investigation, DSS, KP, and TS: Resources, HN, JC, NS, NL, SS, and TS: Data curation, DSS, HN, JC, NS, NL, and TS: Writing – original draft preparation, DSS, TS, and SS: Writing – review and editing, TS: Supervision, TS: Project administration, DSS and TS: Funding acquisition. All authors have read and approved the final manuscript.
